# Moderate Exercise Allows for shorter Recovery Time in Critical Limb Ischemia

**DOI:** 10.3389/fphys.2017.00523

**Published:** 2017-07-25

**Authors:** Anne Lejay, Gilles Laverny, Stéphanie Paradis, Anna-Isabel Schlagowski, Anne-Laure Charles, François Singh, Joffrey Zoll, Fabien Thaveau, Evelyne Lonsdorfer, Stéphane Dufour, Fabrice Favret, Valérie Wolff, Daniel Metzger, Nabil Chakfe, Bernard Geny

**Affiliations:** ^1^Université de Strasbourg, Fédération de Médecine Translationnnelle, Equipe d'Accueil 3072, Mitochondrie, Stress Oxydant et Protection Musculaire, Institut de Physiologie Strasbourg, France; ^2^Service de Physiologie et Explorations Fonctionnelles Respiratoires, Hôpitaux Universitaires de Strasbourg Strasbourg, France; ^3^Service de Chirurgie Vasculaire et Transplantation Rénale, Hôpitaux Universitaires de Strasbourg Strasbourg, France; ^4^Institut de Génétique et Biologie Moléculaire et Cellulaire, Centre National de la Recherche Scientifique UMR7104/Institut National de la Santé et de la Recherche Médicale U964, Université de Strasbourg Strasbourg, France; ^5^Faculté des Sciences du Sport, Université de Strasbourg Strasbourg, France; ^6^Unité Neurovasculaire, Hôpitaux Universitaires de Strasbourg Strasbourg, France

**Keywords:** exercise, ischemia, peripheral arterial disease, mitochondria, muscle, oxidative stress, sarcopenia

## Abstract

Whether and how moderate exercise might allow for accelerated limb recovery in chronic critical limb ischemia (CLI) remains to be determined. Chronic CLI was surgically induced in mice, and the effect of moderate exercise (training five times per week over a 3-week period) was investigated. Tissue damages and functional scores were assessed on the 4th, 6th, 10th, 20th, and 30th day after surgery. Mice were sacrificed 48 h after the last exercise session in order to assess muscle structure, mitochondrial respiration, calcium retention capacity, oxidative stress and transcript levels of genes encoding proteins controlling mitochondrial functions (PGC1α, PGC1β, NRF1) and anti-oxidant defenses markers (SOD1, SOD2, catalase). CLI resulted in tissue damages and impaired functional scores. Mitochondrial respiration and calcium retention capacity were decreased in the ischemic limb of the non-exercised group (V_max_ = 7.11 ± 1.14 vs. 9.86 ± 0.86 mmol 02/min/g dw, *p* < 0.001; CRC = 7.01 ± 0.97 vs. 11.96 ± 0.92 microM/mg dw, *p* < 0.001, respectively). Moderate exercise reduced tissue damages, improved functional scores, and restored mitochondrial respiration and calcium retention capacity in the ischemic limb (V_max_ = 9.75 ± 1.00 vs. 9.82 ± 0.68 mmol 02/min/g dw; CRC = 11.36 ± 1.33 vs. 12.01 ± 1.24 microM/mg dw, respectively). Exercise also enhanced the transcript levels of PGC1α, PGC1β, NRF1, as well as SOD1, SOD2, and catalase. Moderate exercise restores mitochondrial respiration and calcium retention capacity, and it has beneficial functional effects in chronic CLI, likely by stimulating reactive oxygen species-induced biogenesis and anti-oxidant defenses. These data support further development of exercise therapy even in advanced peripheral arterial disease.

## Introduction

Peripheral arterial disease (PAD) is linked to stenosis or occlusion of the arteries that are responsible for decreased perfusion of the lower limbs. PAD symptoms range from intermittent claudication (defined by muscle pain on walking) to critical limb ischemia (CLI; characterized by rest pain or ulcers; Norgren et al., 2007). The clinical and economical impacts of PAD are large, since expensive vascular surgical procedures with high rates of hospitalizations and repeated interventions are required, especially in CLI (Norgren et al., 2007; Mahoney et al., 2010; Shishehbor et al., 2016).

Recently major advances have been observed in the knowledge of PAD pathophysiology (Charles et al., 2017). Notably, interactions between muscle mitochondria and reactive oxygen species (ROS) have been emphasized (Lejay et al., 2014). Oxidative stress, which precedes mitochondrial dysfunction, arises during ischemia, and is enhanced after reperfusion (Guillot et al., 2014). This suggests that modulating ROS production may reduce ischemia-reperfusion injury. Accordingly, mitochondrial function is protected when ROS production is reduced and/or when the production of ROS can be efficiently handled by the antioxidant system (Lejay et al., 2014; Charles et al., 2017). However, interactions between mitochondria and ROS are subtle. Significant increases in ROS production are generally considered to be deleterious because it induces lipid peroxidation, protein carbonylation, and mitochondrial dysfunction that in turn is associated with reduced ATP production (Lejay et al., 2014). Conversely, a slight increase of ROS can act as a signaling pathway that stimulates mitochondrial biogenesis and enhances antioxidant defenses (Charles et al., 2017). Mitochondria are thus largely involved in PAD pathophysiology and they are now considered as a therapeutic target in CLI (Ryan et al., 2015; Paradis et al., 2016).

Exercise therapy represents an effective non-pharmacologic treatment for PAD patients suffering from intermittent claudication by improving walking ability and providing protective effects against mortality in these patients (Gardner et al., 2008; Leeper et al., 2013; Conte and Pomposelli, 2015; Gardner, 2015; Baum et al., 2016; Gouspillou and Hepple, 2016; Whayne and Mukherjee, 2016). Exercise ability also provides prognostic information beyond traditional risk factor assessment since low exercise capacity is strongly related to the risk of mortality in these patients (Parmenter et al., 2015). However, since CLI is an advanced stage of PAD, high intensity-training protocols performed in the setting of intermittent claudication cannot be used (Abe et al., 2012; Lejay et al., 2014; Conte and Pomposelli, 2015). We hypothesized therefore that moderate exercise should be tested in CLI.

We investigated the effects of a specific moderate-intensity exercise-training protocol on CLI symptoms, using our established CLI murine model that mimicks the human pathology (Lejay et al., 2015). Further, since mechanisms involved in the potential beneficial effects of exercise include oxidative stress modulation (Rokutanda et al., 2011; Hoier et al., 2013; Muller et al., 2013; Nunomiya et al., 2016), we have tested the hypothesis that, in this protocol, functional benefits might be linked to restored mitochondrial function and calcium retention capacities which are in turn restored, through of ROS-induced biogenesis and anti-oxidant defenses.

## Materials and methods

### Animals

The study was approved by the CREMEAS ethical committee (AL/70/77/02/13). French laws for animal use and care were respected. Twenty Swiss mice (male, 8 weeks old, weighting 30–35 grams) were handled. Surgery was performed under general anesthesia and post-operative analgesia was obtained using buprenorphine (Buprecare®, Animal care).

### Critical limb ischemia model

General anesthesia was conducted in an airtight ventilated chamber (3% isoflurane (Aerrane®, Baxter Healthcare) and air) and anesthesia maintenance was obtained by spontaneous ventilation through a mask (2% isoflurane, air).

The surgery included two phases. First, the right femoral artery and its first three collateral vessels were ligated under microscope. Ligation of the femoral artery was standardized. It was located midway between the superficial epigastric artery and the bifurcation of the popliteal and saphenous arteries. Second, the right common iliac artery was ligated 0.5 cm distal to its origin, 4 days after the first phase, as described in our established CLI model (Lejay et al., 2015). In this model, the right limb suffers from CLI while the left limb can be considered as control (Thaveau et al., 2007, 2009).

Animals were divided into two groups, one group to be sacrificed 30 days after surgery (Non-exercised group, *n* = 10); and one group to be subjected to exercise and sacrificed 30 days after surgery (Exercised group, *n* = 10).

### Exercise protocol

The exercised group was trained over a 3-week period, five times per week, starting at Day 7 (when CLI was effective). Exercise was performed on a motorized treadmill (Treadmill Control, Letica, Spain). Each session was preceded by a warm-up phase of 2 min (10° incline, 15 cm/s). During Week 1, animals run in the treadmill with a 10° inclination and a belt speed set at 25 cm/s. Length of exercise was 30 min for Day 1 and Day 2, 45 min for Day 3 and 60 min for Day 4 and Day 5. During Week 2, the inclination remains at 10° but the belt speed was set at 30 cm/s. Length of exercise was 60 min for Day 1 and Day 2, 75 min for Day 3 and Day 4 and 90 min for Day 5. During Week 3, animals run for 90 min with the treadmill set at 10° 30 cm/s for Days 1–5. Animals were sacrificed at Day 30 (48 h after the last exercise session).

### *In vivo* clinical follow-up

*In vivo* clinical follow-up was assessed using already-established clinical scores (Stabile et al., 2003). Tissue damage was graded attributing 1 for a normal aspect of the limb, 2 for a white aspect, 3 for toe cyanosis, 4 for necrosis, and 5 for spontaneous amputation of a toe. Functional score was graded attributing 0 for a normal function of the limb, 1 for plantar flexion without toe flexion, 2 when no plantar flexion could be achieved and 3 when the mouse dragged the limb (Lejay et al., 2014). Clinical scores were assessed at Day 0, 4, 6, 10, 20, and 30 for all animals by an independent evaluator.

### *Ex vivo* muscle analysis

All animals were sacrificed at Day 30. Ischemic and contralateral gastrocnemius muscles were collected. The activity of mitochondrial respiration chain complexes, calcium retention capacity and production of free radicals were immediately assessed. Tibialis muscles were immersed in liquid nitrogen and stored at −80°C, for subsequent histological and RNA transcripts analysis.

#### Mitochondrial respiratory chain complex activities

Mitochondrial respiratory chain involves four complexes producing energy by electron transfers and oxygen consumption. In order to study the activity of mitochondrial respiratory chain complexes the consumption of oxygen in skinned skeletal muscle fibers is measured. This allows for a reliable assessment of the functional oxidative capacity (Charles et al., 2011, 2017; Mansour et al., 2012; Talha et al., 2012). Oxygen consumption is measured with Clark electrodes (Strathkelvin Instruments®, United Kingdom). A difference of potential of 0.6–0.7 mV is applied between the anode (silver electrode) and the cathode (platinum electrode), both immersed in a KCl solution. The intensity of the induced current, a recordable signal, is related to the conductance between both electrodes via the KCl bridge and is directly proportional to the oxygen consumption from the medium. Slopes of the signal, which are also measured, reflects the rate of oxygen consumption (V0). After V0 determination, substrates were added in order to specifically activate or inhibit the respiratory complexes. ADP (2 mM) and Succinate (25 mM) are then added to study complexes I, II, III, and IV activity, which determines the maximal oxidative capacity: Vmax. The addition of Amytal (0.02 mM) subsequently inhibits complex I, allowing determining Vamytal (complexes II, III, and IV activities). The addition of N, N, N′, N′-tetramethyl-p-phenylenediamine dihydrochloride (TMPD 0.5 mM) and ascorbate (0.5 mM) specifically activates complex IV (Vtmpd; Charles et al., 2011; Mansour et al., 2012).

#### Calcium retention capacity

Calcium retention capacity of fibers from gastrocnemius muscle fibers is measured by spectrofluorometry. This capacity is defined as the high cut-off amount of calcium that determines the opening of the mitochondrial transition pore, which allows the release of calcium that will in turn lead to apoptosis. Calcium pulses (20 μM) were applied to skinned fibers of gastrocnemius muscle. The mitochondrial calcium uptake was thereafter assessed after the addition of a single calcium pulse (20 μM), by measuring the decrease of the extramitochondrial calcium concentration. The measure was monitored using a fluorescent probe (Calcium Green-5N, Invitrogen). When mitochondrial calcium release started, that is when the calcium uptake showed an inflection point, it was considered that mPTP opening was observed. Mitochondrial calcium retention capacity, which is a reliable index of mPTP sensitivity, was calculated as the total amount of calcium taken up by mitochondria before calcium release.

#### Production of reactive oxygen species using electron paramagnetic resonance

Gastrocnemius muscles (1 mm3 fragments) were incubated with a 200 μM CMH molecular probe (1-hydroxy-3-methoxycarbonyl-2, 2, 5, 5-tetramethylpyrrolidine HCl), which is oxidized in the presence of unpaired electrons of ROS. The amount of oxidized CMH, and thus the related amount of free radicals produced, was measured by the intensity of the resonance signal (Noxygen®, Allemagne).

#### Histological analysis: muscle structure and ROS production

Tibialis muscles were immersed in liquid nitrogen and stored at −80°C. Muscles were embedded in paraffin. A cryostat microtome was then used in order to obtain 10 mm thick sections which were mounted onto glass slides. Hematoxylin and eosin stain was applied to the specimens which were examined under bright-field microscopy. Specimens were also incubated with 2.5 mM dihydroethidium (DHE), which produces red fluorescence when oxidized to ethidium bromide (mainly by superoxide anion). After staining, sections were examined under epifluorescence microscope (Nikon Eclipse E800) and emission signals recorded.

#### RNA transcripts encoding for mitochondrial biogenesis and antioxidant defense

Tibialis muscles were immersed in liquid nitrogen and stored at −80°C. Transcripts encoding antioxidative enzymes (SOD1, SOD2, catalase) and proteins involved in mitochondrial function (PGC1α, ß, and NRF1) were analyzed. Two micrograms of RNA, isolated with TRIzol Reagent (Invitrogen), were converted to cDNA with SuperScript II reverse transcriptase (Invitrogen, Life Technologies) and hexamer primers according to the supplier's protocol. Quantitative RT-PCR was performed using the QuantiTectTM SYBR Green PCR kit (Roche) according to the supplier's protocol. Primers were for Sod1, 5′-CCAGTGCAGGACCTCATTTT-3′ (sense) and 5′-TTGTTTCTCATGGACCACCA-3′ (antisense); for Sod2, 5′-ACCCAAAGTCACGCTTGATAG-3′ (sense) and 5′-GGACAAACCTGAGCCCTAAG-3′ (antisense); for Catalase, 5′-CACTGACGAGATGGCACACT-3′ (sense) and 5′-TGTGGAGAATCGAACGGCAA-3′ (antisense); for Pgc-1α, 5′-AAGTGTGGAACTCTCTGGAACTG-3′ (sense) and 5′-GGGTTATCTTGGTTGGCTTTATG-3′ (antisense); for Pgc-1β, 5′-TGCGGAGACACAGATGAAGA-3′ (sense) and 5′-GGCTTGTATGGAGGTGTGGT-3′ (antisense); for Nrf-1, 5′-TGGAGTCCAAGATGCTAATG-3′ (sense) and 5′-AGAGCTCCATGCTACTGTTC-3′ (antisense).

### Data analysis

Statistical analysis was performed with GraphPad Prism 5® (GraphPad Software, Inc.) under the supervision of the Department of Statistics of our university. Clinical follow-up scores of groups were compared using *t*-tests. Other parameters were compared using a one-way ANOVA design.

Results are expressed as means and standard deviations. *p* < 0.05 were considered as indicative of statistical significance.

## Results

### Functional and muscular effects of chronic limb ischemia

#### *In vivo* clinical follow up

**Figure 1:**
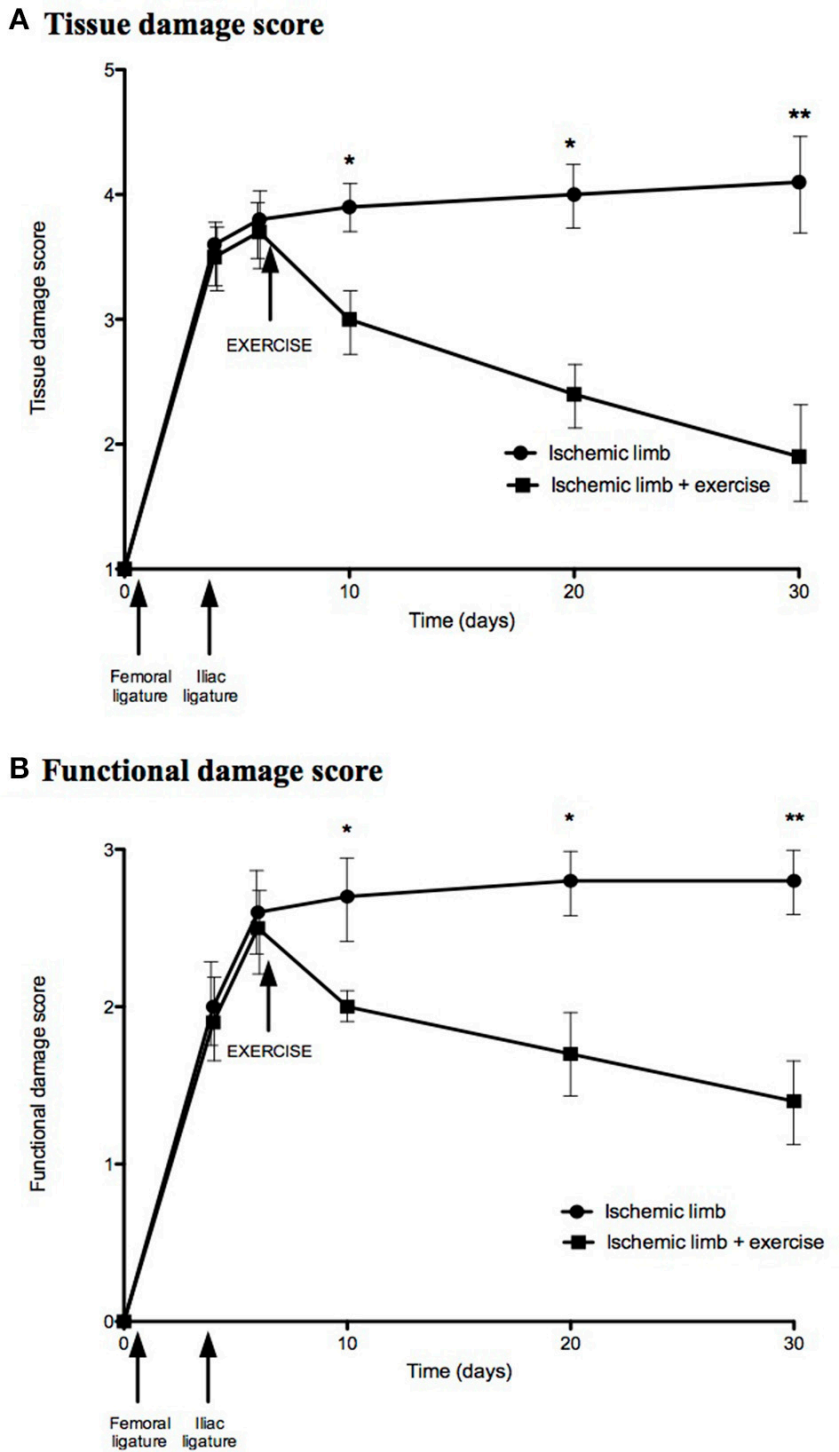
Exercise improved tissue damage and functional scores in the exercised group compared to the non-exercised group. Tissue damage **(A)** and functional **(B)** scores were evaluated in non-exercised (*n* = 10) and exercised (*n* = 10) groups. Results are expressed as means and standard deviations at days 0, 4, 6, 10, 20, and 30. ^*^p < 0.05; ^**^p < 0.01.

At Day 30, among the 10 mice in the non-exercised group, ischemic limb showed auto-amputation of a toe in two mice, toe necrosis in seven mice and toe cyanosis in one mouse. Mean tissue damage score increased progressively from Day 4 to Day 30 with scores of 3.6, 3.8, 3.9, 4.0, and 4.1 at Days 4, 6, 10, 20, and 30, respectively. Concerning limb function, 8 mice dragged the ischemic limb and 2 could not achieve plantar flexion at Day 30. Mean functional damage score increased progressively from Day 4 to Day 30 with scores of 2.0, 2.6, 2.7, 2.8, and 2.8 at Days 4, 6, 10, 20, and 30, respectively (Figure 1). Contralateral limbs showed no tissue or functional damage.

#### *Ex-vivo* analysis

**Figure 2:**
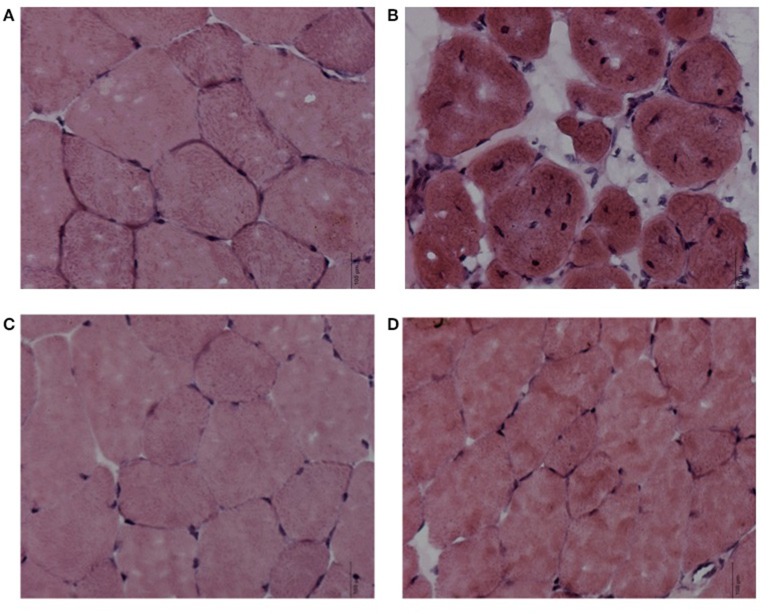
Exercise allowed the recovery of the myopathic feature. Tibialis muscles specimens were examined under bright-field microscopy after hematoxylin-eosin stain (x40). Hematoxylin eosin showed myopathic features of ischemic muscles in the non-exercised group (**A** Control limb, **B** Ischemic limb), exercise does not (**C** Control limb + exercise, **D** Ischemic limb + exercise).

In agreement with previous results (Lejay et al., 2015), hematoxylin eosin staining showed typical myopathic features of ischemic muscles, with wider range in fiber size, a more rounded shape, centrally located nuclei, and smaller cross-sectional areas compared to control fibers (Figure 2).

**Figure 3:**
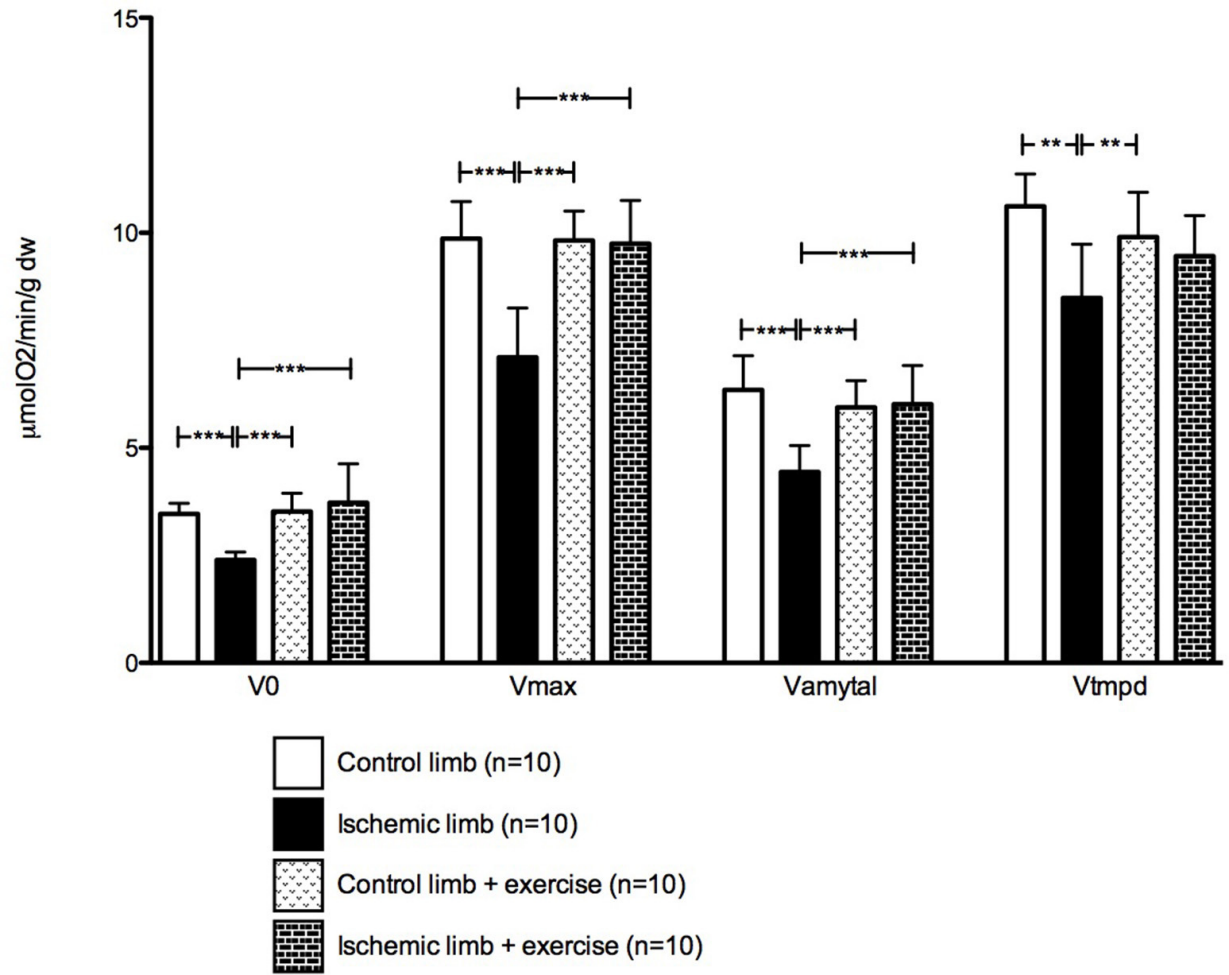
Mitochondrial respiration is restored in ischemic limbs after exercise. V_0_ = Basal mitochondrial oxidative capacity; V_max_ = Maximal mitochondrial oxidative capacity; V_amytal_ = Complexes II, III, and IV activity; V_tmpd_ = Complexes IV activity. Mean ± SD at Day 30 in the non-exercised group (*n* = 10) and in the exercised group (*n* = 10). ^**^p < 0.01; ^***^p < 0.001.

Mitochondrial respiration was decreased in chronic ischemic muscles. V0 was 3.47 ± 0.24 mmol 02/min/g dry weight (dw) in control limb and 2.39 ± 0.19 mmol 02/min/g dw in ischemic limb (*p* < 0.001). The same applied to Vmax (9.86 ± 0.86 in control limb vs. 7.11 ± 1.14 mmol 02/min/g dw in ischemic limb, *p* < 0.001); Vamytal (6.35 ± 0.79 in control limb vs. 4.44 ± 0.62 mmol 02/min/g dw in ischemic limb, *p* < 0.001); and Vtmpd (10.62 ± 0.76 in control limb vs. 8.49 ± 1.25 mmol 02/min/g dw in ischemic limb, *p* < 0.001; Figure 3).

**Figure 4:**
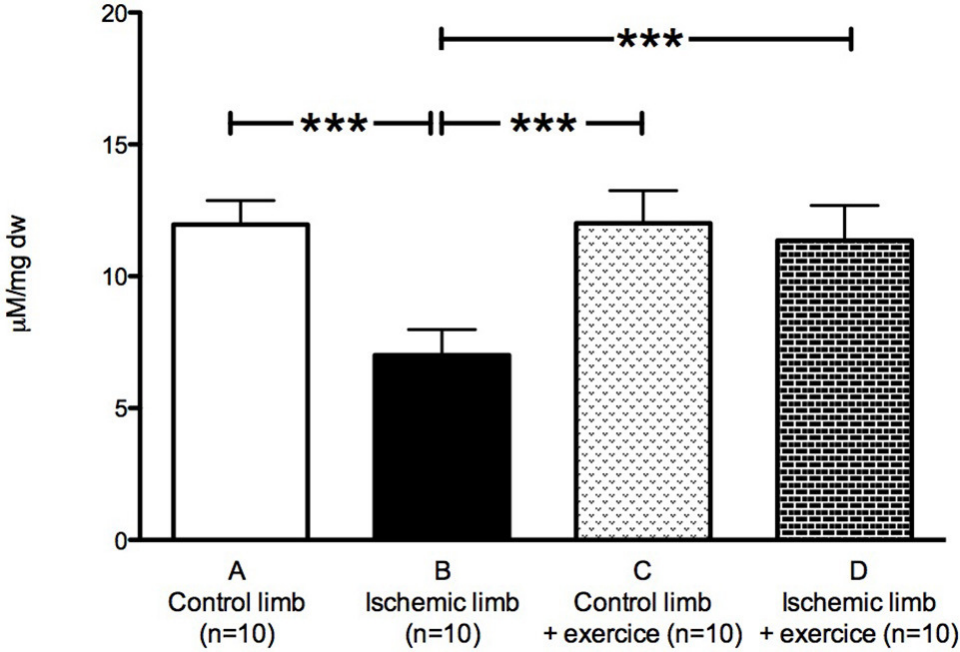
Calcium retention capacity is restored in ischemic limbs after exercise. Mean ± SD at Day 30 in the non-exercised group (*n* = 10) and in the exercised group (*n* = 10). ^***^p < 0.001.

Calcium retention capacity was impaired in ischemic limbs: 11.96 ± 0.92 in control limbs vs. 7.01 ± 0.97 μM/mg dw in ischemic limbs (*p* < 0.001; Figure 4).

**Figure 5:**
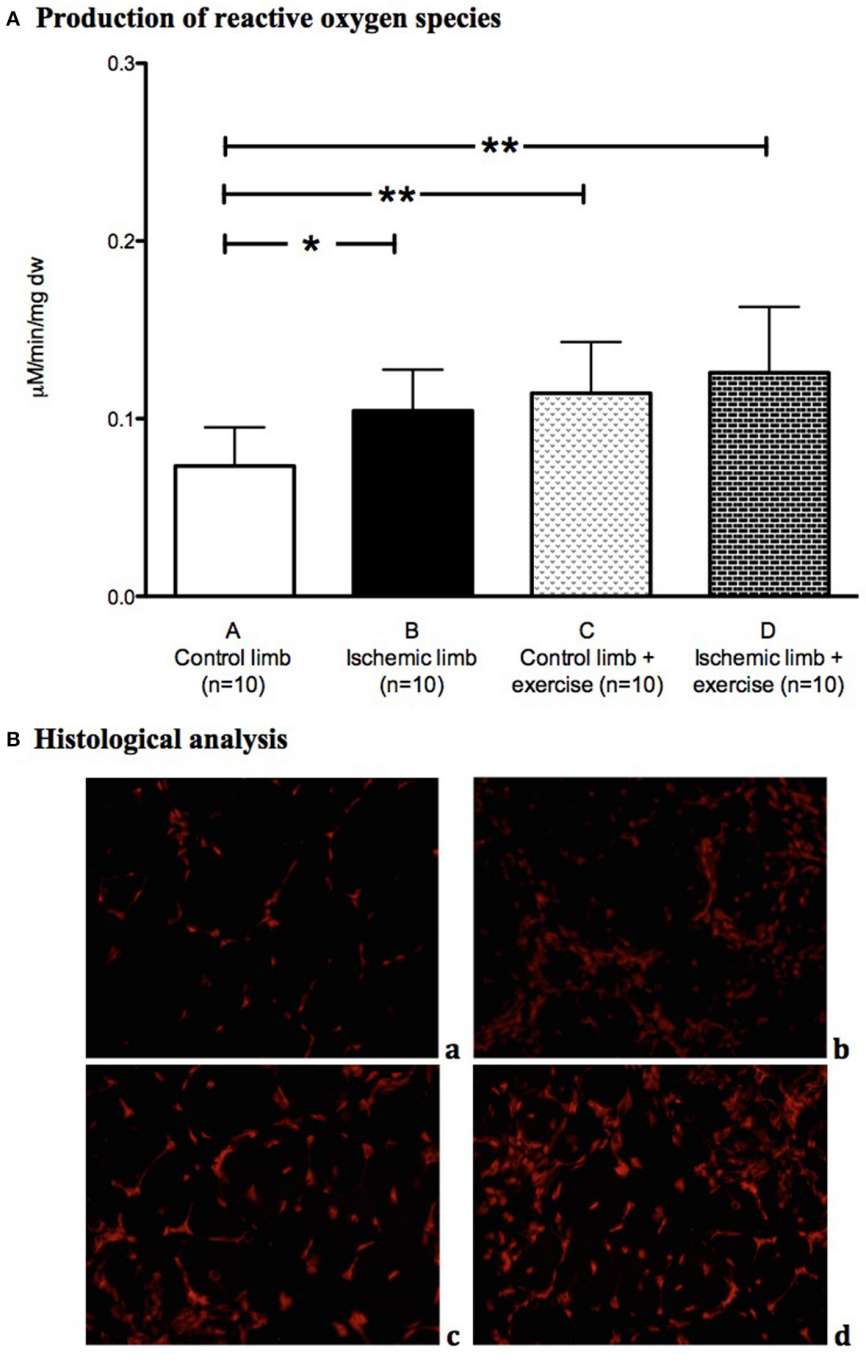
Oxidative stress is increased by critical limb ischemia and exercise. Production of free radicals **(A)** was increased in ischemic muscles (*n* = 10) compared to control muscles (*n* = 10) in the non-exercised group. DHE staining **(B)** showed higher fluorescence in ischemic muscles compared to control muscles (**a**, Control limb; **b**, Ischemic limb) in the non-exercised group. Production of free radicals was increased in the exercised group compared to the non-exercised group **(A)**. Exercice showed higher fluorescence **(B)** in control and ischemic limbs (**c**, Control limb + exercise; **d**, Ischemic limb + exercise). ^*^p < 0.05; ^**^p < 0.01.

Production of free radicals was significantly increased in ischemic muscles (0.11 ± 0.02 mmol/min/mg dw) compared to control limbs (0.07 ± 0.02 mmol/min/mg dw; *p* < 0.05; Figure 5A). DHE staining showed higher fluorescence in ischemic limbs compared to control limbs (Figure 5B).

**Figure 6:**
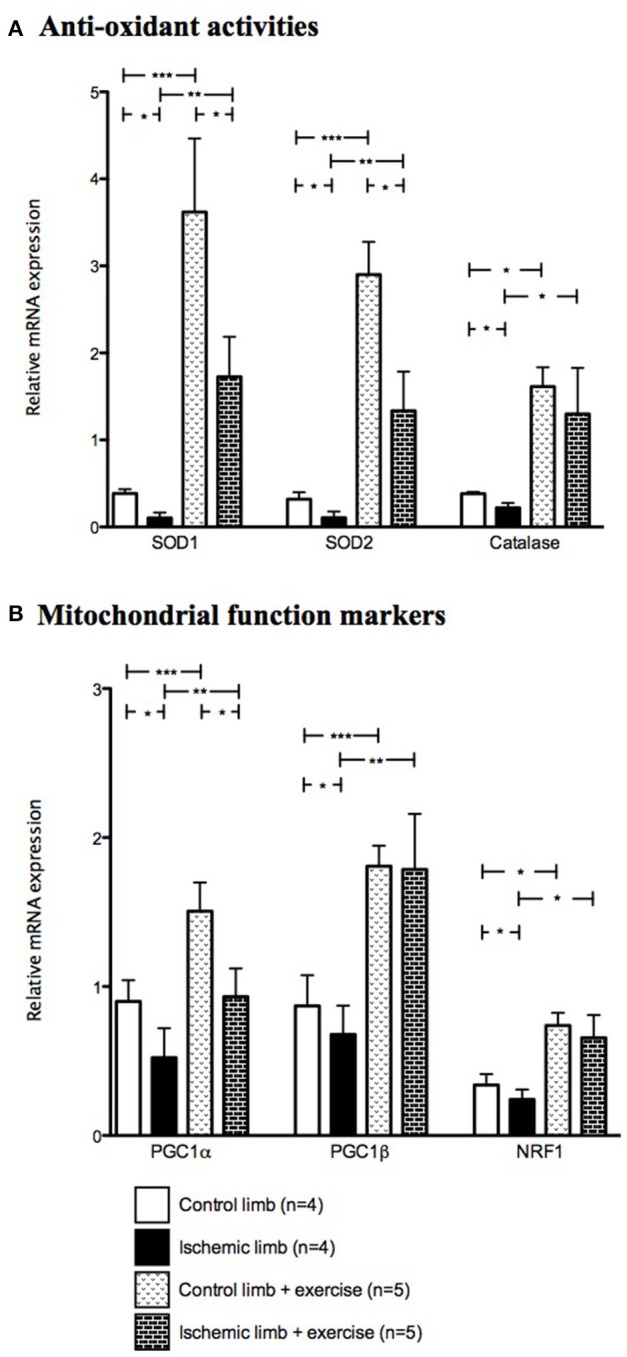
Relative mRNA expression of anti-oxidant and mitochondrial function markers are increased after exercise. Mean ± SD at Day 30 in the non-exercised group (*n* = 4) and in the exercised group (*n* = 5). ^*^p < 0.05; ^**^p < 0.01; ^***^p < 0.001. (SOD, superoxide dismutase; PGC, proliferator activated receptor gamma coactivator; NRF, nuclear respiratory factor).

Finally, ischemia decreased mRNA expression of antioxidant enzymes (0.39 ± 0.10 vs. 0.10 ± 0.06 for SOD1 (*p* < 0.05), 0.32 ± 0.16 vs. 0.11 ± 0.07 for SOD2 (*p* < 0.05) and 0.38 ± 0.04 vs. 0.22 ± 0.11 for catalase (*p* < 0.05) in control and ischemic muscles respectively; Figure 6A). Ischemia also decreased the relative mRNA expression of mitochondrial function markers (0.90 ± 0.29 vs. 0.52 ± 0.39 for PGC1α (*p* < 0.05), 0.87 ± 0.41 vs. 0.68 ± 0.39 for PGC1β (*p* < 0.05) and 0.34 ± 0.14 vs. 0.24 ± 0.13 for NRF1 (*p* < 0.05) in control and ischemic muscles respectively; Figure 6B).

### The deleterious effects of chronic limb ischemia are attenuated by moderate exercise

#### *In vivo* clinical follow up

After ischemia, among the 10 mice in exercised group, one mouse presented with toe cyanosis, seven mice had a white aspect of the limb, and two mice had a normal aspect of the limb at Day 30. Mean tissue damage decreased after exercise initiation from Day 4 to Day 30 with scores of 3.5, 3.7, 3.0, 2.4, and 1.9 at Days 4, 6, 10, 20, and 30, respectively. Tissue damage was significantly decreased in exercised group compared to non-exercised group.

At Day 30, four mice could not achieve plantar flexion, and six mice could not achieve toe flexion. Mean functional damage score decreased after exercise initiation from Day 4 to Day 30 with scores of 1.9, 2.5, 2.0, 1.7, and 1.4 at Days 4, 6, 10, 20, and 30, respectively (Figure 1). Functional damage was significantly decreased in exercised group compared to non-exercised group.

### Contralateral limbs showed no tissue or functional damage

#### *Ex-vivo* analysis

Unlike muscles without exercise, exercised ischemic muscles did not exhibit myopathic features (Figure 2).

Mitochondrial respiration was restored in ischemic muscles, since it was not significantly different in control limbs as compared to ischemic limbs (V0 was 3.52 ± 0.42 vs. 3.73 ± 0.90 mmol O2/min/g dw, Vmax was 9.82 ± 0.68 vs. 9.75 ± 1.00 mmol O2/min/g dw, Vamytal was 5.94 ± 0.62 vs. 6.02 ± 0.90 mmol O2/min/g dw, and Vtmpd was 9.90 ± 1.04 vs. 9.46 ± 0.95 mmol O2/min/g dw in control and ischemic limbs respectively; Figure 3).

Calcium retention capacity was restored in ischemic muscles: 12.01 ± 1.24 in control exercised limbs vs. 11.36 ± 1.33 μM/mg dw in ischemic exercised limbs (Figure 4).

Free radicals production showed no significant difference between control exercised muscles and ischemic exercised muscles: 0.11 ± 0.03 vs. 0.13 ± 0.04 mmol/min/mg dw respectively (Figure 5A). DHE staining exhibited a significantly higher fluorescence in exercised muscles compared to non-exercised muscles, even in control limbs (Figure 5B).

Exercise increased significantly mRNA levels of anti-oxidant enzymes in control muscles [0.39 ± 0.10 vs. 3.62 ± 1.89 for SOD1 (*p* < 0.001), 0.32 ± 0.16 vs. 2.90 ± 0.84 for SOD2 (*p* < 0.001) and 0.39 ± 0.16 vs. 1.61 ± 0.49 for catalase (*p* < 0.05) in non-exercised and exercised muscles respectively]. Exercise also increased significantly the transcripts of anti-oxidant enzymes in ischemic muscles (0.10 ± 0.06 vs. 1.73 ± 1.03 for SOD1 (*p* < 0.01), 0.11 ± 0.07 vs. 1.33 ± 1.01 for SOD2 (*p* < 0.01) and 0.22 ± 0.11 vs. 1.29 ± 1.18 for catalase (*p* < 0.05) in non-exercised and exercised muscles respectively; Figure 6A).

Exercise also increased significantly mRNA levels of mitochondrial function markers in control muscles [0.90 ± 0.29 vs. 0.52 ± 0.39 for PGC1α (*p* < 0.05), 0.87 ± 0.41 vs. 1.81 ± 0.31 for PGC1β (*p* < 0.001), and 0.34 ± 0.14 vs. 0.74 ± 0.19 for NRF1 (*p* < 0.05) in non-exercised and exercised muscles respectively], as well as in ischemic muscles [0.52 ± 0.39 vs. 0.93 ± 0.42 for PGC1α (*p* < 0.01), 0.68 ± 0.39 vs. 1.79 ± 0.83 for PGC1β (*p* < 0.01) and 0.24 ± 0.13 vs. 0.66 ± 0.34 for NRF1 (*p* < 0.05; Figure 6B)]. Thus, even though PGC1α, PGC1β, NRF1, SOD1, SOD2, and catalase transcript levels were lower in ischemic limb muscles than in control muscles of non-exercised mice, they were similar or higher in exercised ischemic limb muscle than in non-exercised control limb.

## Discussion

The key findings of this study are that moderate exercise, which slightly increases oxidative stress, might allow for shorter time for limb recovery, probably by restoring functional capacities, mitochondrial function and calcium retention capacity in the setting of CLI. It is likely that exercise induces an increased mitochondrial biogenesis, as well as antioxidant capacities in skeletal muscles, which may involve ROS signaling. To our knowledge, this is the first study suggesting the beneficial role of low-intensity exercise in the setting of CLI.

### Chronic critical limb ischemia characteristics

This model fulfills all criteria defining CLI, since the use of sequential artery ligations induces chronic hypoperfusion (confirmed by scintigraphies) responsible for clinical and functional symptoms lasting for more than 2 weeks (Norgren et al., 2007; Lejay et al., 2015). Previous models of lower limb ischemia are mostly based on a single arterial ligation, mainly a femoral artery (just distal to the origin of the *profunda femoris*) ligation (Lofti et al., 2013). However, this method leaves most of the collateral circulation to the lower limb intact, and consequently, blood flow to the limb is always restored within 7 days. Another surgical model is based on a total excision of the femoral artery, including the collateral vessels. However, blood flow is restored progressively. This can be explained since the collateral bed mostly arises from the internal iliac artery (Lofti et al., 2013). In our CLI model, we performed sequential ligations. This allowed us to obtain a sustainable hypoperfusion, likely because the second ligation was performed on the common iliac artery and reduced the collateral perfusion provided by the internal iliac artery (Lejay et al., 2015). Moreover, even if ligation of the blood flow of one limb might produce hormonal changes systematically, we already showed that contralateral limb can be considered a control (Lejay et al., 2015).

### Beneficial effects of moderate exercise in chronic critical limb ischemia

Preclinical or clinical studies examining the effect of exercise in the setting of intermittent claudication are numerous (Gardner et al., 2008; Abe et al., 2012; Haas et al., 2012; Muller-Buhl et al., 2012; Hoier et al., 2013), but few data are available in the setting of CLI, despite its increasing occurrence and morbidity (Olin et al., 2016). Thus, outcomes are worse in patients with CLI: at 1 year, 10% of them will undergo a deadly cardiovascular event, and 25% of them will require leg or thigh amputation (Norgren et al., 2007; Olin et al., 2016).

Exercise therapy is proposed in first intention for patients presenting with infrainguinal lesions and intermittent claudication (Norgren et al., 2007; Daussin et al., 2008; Da Silva et al., 2015; Baum et al., 2016), but, since CLI patients can be physically limited and thus restricted in terms of walking capacities or exercise tolerance, such a therapy is currently not used (Muller-Buhl et al., 2012; Baum et al., 2016; Olin et al., 2016). We therefore specifically aimed at studying the effects of a moderate-intensity training protocol.

Our data demonstrate that moderate-intensity exercise training reduces significantly tissue damages. Indeed, confirming the functional improvement observed after exercise, HE staining highlights the fact that the CLI-related myopathic features disappear after training.

Further analyzing skeletal muscle integrity, we also observed a normalization of the mitochondrial respiration; all complexes of the mitochondrial respiratory chain showing increased oxidative capacities. This is a key result since, besides reduced oxygen supply, mitochondrial dysfunction participates in CLI pathophysiology (Dopheide et al., 2013; Lejay et al., 2014, 2015; Chen et al., 2016) and accordingly, improvement in muscle mitochondrial function has been shown to be associated with improved prognosis in PAD patients (Pedersen et al., 2009).

In addition, we observed that moderate exercise restored the mitochondrial calcium retention capacity, delaying mPTP opening and therefore likely apoptosis. Indeed, the mPTP is a non-selective channel located through the inner mitochondrial membrane which is predominantly in a closed state during physiological conditions. A major consequence of mPTP opening is dissipation of the proton electrochemical gradient, leading to the inhibition of ATP production and finally, mitochondrial swelling, and rupture, initiating the apoptotic processes (Tran et al., 2012; Pottecher et al., 2013; Garbaisz et al., 2014). Some experimental studies examined the effect of acute lower-limb ischemia-reperfusion on mPTP (Tran et al., 2012; Pottecher et al., 2013; Garbaisz et al., 2014), but this is the first work investigating the potential modulating effect of exercise on mPTP opening during CLI.

Interestingly, moderate exercise delays mPTP opening, mitigating therefore CLI damages, and favoring increased functional abilities. This highlights the potential of exercise therapy in CLI, particularly knowing that improved exercise performance generally results in quality of life improvement and thus better prognosis.

Exercise could be achieved despite flow limitation and mitochondrial function impairment. Indeed, one might suggest that strong flow limitation should be associated with “no” exercise capacity. In fact, although significant, mitochondrial impairment is relatively moderate and our model induces hypoperfusion rather than complete ischemia. Additionally, muscle susceptibility to ischemia reperfusion depends on their metabolic phenotype and thus some muscles—mainly oxidative—might be less impaired (Charles et al., 2017). Compensation by other muscles that are not or only slightly impaired by ischemia reperfusion might play a role in the degree of functional impairment observed after ischemia reperfusion injury.

### Mechanisms likely involved in exercise-related improvements

We aimed to challenge the hypothesis that ROS signaling might participate in muscle protection through mitochondrial functions and anti-oxidative pathways enhancement. Although, ROS production is thought to be a key event during ischemia-reperfusion-induced deleterious effects, its kinetic is poorly studied and particularly, whether there is ROS production during ischemia remains a debated question (Paradis et al., 2016). Indeed, lack of oxygen availability during ischemia should preclude ROS formation, and models that report ROS production in ischemia may take oxygen from the room air. However, we previously demonstrated that ROS generation occurs during ischemia (Guillot et al., 2014). Several hypotheses might be proposed. First, hypoxia might not be total because of residual O2 levels in the tissues. Additionally, our CLI model does not induce complete ischemia, but rather hypoperfusion and therefore hypoxemia. Finally, a reduced antioxidant defense secondary to ischemia might explain an increased ROS level during ischemia even if production is only slightly enhanced.

It has been demonstrated that exercise induces ROS production in skeletal muscle (Sakellariou et al., 2014; Merry and Ristow, 2016). The conjunction of CLI-induced ROS production and exercise-induced ROS production might favor mitochondrial dysfunction. However, exercise might contribute to ameliorate mitochondrial function despite increased oxidative stress, if ROS production is low enough to result in a positive biological signal, so called “mitohormesis.” Hormesis is a process whereby exposure to low stress level promotes adaptive changes allowing the cell to better tolerate subsequent greater stress (Bouitbir et al., 2012; Lejay et al., 2014; Merry and Ristow, 2016). Strong evidence suggests that mitochondria allow the initiation and transduction of a signal to the nucleus, resulting in a transcriptional response with both mitochondrial and non-mitochondrial adaptations, maintaining cellular homeostasis (Ji et al., 2006, 2016).

Our results suggest that ROS signaling is, at least in part, responsible for mediating exercise-induced increases in mitohormesis within skeletal muscle, as well as antioxidant capacity (He et al., 2016; Ji et al., 2016; Merry and Ristow, 2016). Indeed, although excessive ROS production is damaging the cardiovascular system, small amounts of ROS are beneficial for adaptive processes following ischemia (Ebrahimian et al., 2006; Haas et al., 2012). Particularly, ROS have been shown to play the role of important cell signaling molecules when analyzing beneficial exercise-induced adaptations to skeletal muscle (Irrcher et al., 2009). Accordingly, exercise is much less beneficial when exercise-induced ROS cell signaling is decreased, either by enzymatic inhibitors or antioxidant supplementation (Ljubicic et al., 2010; Higashida et al., 2011; Meier et al., 2013; Wadley et al., 2013; Strobel et al., 2014; He et al., 2016; Ji et al., 2016; Merry and Ristow, 2016; Boveris and Navarro, 2008). In our study, we postulate that mitohormesis and activation of the antioxidant machinery might be contributing to the signaling of exercise-related adaptation in CLI.

Accordingly, exercise increases the expression of PGC1-α and NRF1. PGC1-α is considered as one of the most important regulator of mitohormesis. Specifically in skeletal muscle, a large body of evidence suggests that PGC1-α regulates mitochondrial function, mitochondrial respiration and biogenesis. In particular, PGC1-α interacts with NRF-1 to activate genes involved in the respiratory chain and to stimulate mitochondrial biogenesis (Ljubicic et al., 2010).

Similarly, PGC1-β was shown to promote the expression of genes involved in mitochondrial structure and function, but also to be significantly involved in anti-oxidant defense (Charles et al., 2013; Gali Ramamoorthy et al., 2015; Lee et al., 2016). Further, decreased PGC1-β levels in aged muscle correlates with mitochondrial function decline (Charles et al., 2013). In our study, exercise increased mRNA expression of PGC1-β and anti-oxidant enzymes SOD-1, SOD-2 and catalase, suggesting that the beneficial effect of moderate exercise might be, at least partly, related to muscle antioxidant capacity enhancement.

This study presents several limitations. Clearly, moderate exercise promotes recovery of the ischemic limb in CLI, but experiments of contraction strength would have been useful to further assess muscle function. Moreover, our data do not allow differentiating between increased mitochondrial number and/or capacities. Finally, further studies at the protein levels should further reinforce the implication of mitochondria and ROS in the mechanisms involved.

In conclusion, moderate exercise training in CLI provides beneficial effects allowing accelerated limb recovery. Mechanisms involved in this protective effect could be activation of antioxidant defenses and mitohormesis, supporting that moderate exercise should be taken into account as a potential therapeutic option in patients with CLI.

## Author contributions

AL: Study design, data collection, data analysis, writing. GL, SD: Data analysis. SP, AS, FS, EL, VW: Writing. AC: Data collection, data analysis. JZ: Study design, data collection. FT, FF: Study design. DM: Data analysis, writing. NC: Study design, writing. BG: Study design, data analysis, writing.

### Conflict of interest statement

The authors declare that the research was conducted in the absence of any commercial or financial relationships that could be construed as a potential conflict of interest.
